# Pneumaturia

**Published:** 1915-11

**Authors:** 


**Pneumaturia.** The Jour. d'Urologie reports a case in a man aged 32, due to polymorphic, aerogenic, gram-negative bacilli. The last few drops of urine were passed with a gurgling sound. The urine was acid, contained much indican and some albumin. The sediment contained leucocytes and some red cells.

